# Revision of the Genus *Laelius* (Hymenoptera, Chrysidoidea, Bethylidae) from China

**DOI:** 10.3390/insects15080627

**Published:** 2024-08-20

**Authors:** Chunhong Wang, Junhua He, Xuexin Chen

**Affiliations:** 1 Ministry of Agriculture Key Laboratory of Molecular Biology of Crop Pathogens and Insects, Zhejiang University, Hangzhou 310058, China; joycek0324@163.com; 2 Institute of Insect Sciences, College of Agriculture and Biotechnology, Zhejiang University, Hangzhou 310058, China; 3 Zhejiang Provincial Key Laboratory of Biology of Crop Pathogens and Insects, Zhejiang University, Hangzhou 310058, China; 4 State Key Laboratory of Rice Biology, Zhejiang University, Hangzhou 310058, China

**Keywords:** Epyrinae, new species, new records, DNA barcodes, key

## Abstract

**Simple Summary:**

The cosmopolitan genus *Laelius* Ashmead belongs to the subfamily Epyrinae and includes 68 valid species. Members of *Laelius* mainly attack larvae of larder beetles (Coleoptera, Dermestidae), which are commonly known as pests of stored products. The taxonomic study of *Laelius* in China is far from enough, with only two species recorded. Taking account of the promising biocontrol agents for the pests of forest and stored products, the Chinese species of *Laelius* are revised in this study. Morphological characters and DNA barcoding with COI sequences are used for species delimitation. A new species, *Laelius longus* sp. nov., is described and illustrated. Three new records from China are updated.

**Abstract:**

The genus *Laelius* from China is revised for the first time and six species are recognized, including one new species as well as three new records. The new species, *Laelius longus* sp. nov., which is supported by both morphological and molecular analyses, is described and illustrated. Three new records, *L. naniwaensis*, *L. nigrofemoratus*, and *L. yamatonis*, are illustrated. A key to the Chinese species of *Laelius* is provided.

## 1. Introduction

The genus *Laelius* Ashmead, 1893, belonging to the subfamily Epyrinae, can be easily recognized from other genera by having the body and wings with black thick setae, mesoscuto-scutellar sulcus evident and usually smooth [1]. Species of *Laelius* mostly are ectoparasitoids of dermestid larvae, which include many important stored products’ pests (e.g., *Anthrenus flavipes* which is a destructive pest of natural history specimens, *Trogoderma granarium* which is a voracious feeder of stored products and is listed as one of the 100 worst invasive species worldwide) [2,3]. Therefore, this genus has attracted the attention of applied entomologists for potential application in biological control.

In a taxonomic revision of *Laelius* from the Neotropical region, Barbosa and Azevedo [4] described seven new species and produced a key to males of Neotropical *Laelius* based on genitalia for the first time. Marques et al. [5] published a systematic revision of Afrotropical *Laelius* and provided a list of world species. *Laelius* currently consists of 65 extant species and three fossil species. Twenty-six species have been recorded from the Palaearctic region and four species have been recorded from the Oriental region. Only two species, *Laelius sinicus* and *Laelius jilinensis*, have been recorded from China [6,7]. *Laelius sinicus* has been recorded from Eastern China, while *L. jilinensis* has been recorded from Northeast China. Considering the wide potential habitat of *Laelius* species in China, a further taxonomic study on this genus is needed.

In this study, we examined the Chinese specimens of *Laelius*. DNA barcoding with COI sequences through two different molecular species delimitation methods (ABGD and PTP) are performed to investigate the intra- and interspecific variation within *Laelius*. The new species, *Laelius longus* sp. nov., is supported by our DNA barcoding analyses. Finally, six species, including one new species and three new records, are recognized and a key to all Chinese species is provided.

## 2. Materials and Methods

### 2.1. Specimens

The specimens examined in this study were collected through the sweeping net, Malaise traps, or rearing from host larvae. All the specimens examined in this study are deposited in the Parasitic Hymenoptera Collection of Zhejiang University, Hangzhou, China (ZJUH).

### 2.2. Preparation for Morphological Study

Male genitalia were dissected using an apical curved micro insect pin; subsequent cleaning of hypopygium and genitalia was performed according to the method by Martinelli et al. [8]. The dissected hypopygium and the genitalia were stored in microtubes with glycerin.

Morphological terminology follows Lanes et al. [9] and Brito et al. [10]. The following abbreviations are used for morphological terms and biometric measurements: AOL = width between anterior and posterior ocellus, measured as minimum length in antero-dorsal view; LH = length of head, measured in antero-dorsal view, from apex of clypeus to vertex; OOL = shortest distance from a posterior ocellus to nearest eye margin; POL = posterior ocellus line, measured as minimum width between posterior ocelli in antero-dorsal view; WF = width of frons, measured in antero-dorsal view, its minimum width; WH **=** width of head, measured in antero-dorsal view, its maximum width including eyes; WOT = width of ocellar triangle, measured in antero-dorsal view, maximum width including ocelli. 

A Nikon stereomicroscope (SMZ800N) (Nikon Corporation, Tokyo, Japan) was used for observation. The biometric measurements and the photos of the external and genitalia characters were taken through the digital microscope Keyence (VHX-7000) (Keyence Corporation, Osaka, Japan). The photos were partly enhanced and laid out on a plate using Adobe Photoshop 2023.

### 2.3. Molecular and Phylogenetic Analyses

For the species delimitation analyses, the available *Laelius* COI sequences in BOLD and Genbank were searched, and 96 sequences of *Laelius* and a single sequence of *Gracilepyris rufipes* representing the outgroup were downloaded (Table S2). In addition to this dataset, a single sequence of *Laelius* from China (GenBank accession #PP994884) was obtained in this study by using the modified QIAamp DNA Mini Kit (Qiagen, Hilden, Germany) protocol, the nondestructive method for DNA extraction as suggested by Ayana et al. [11]. Briefly, a freeze–thaw step was added at the start of the extraction to enhance sample DNA release. Specifically, the sample was incubated with 180 μL Buffer ATL and 20 μL at 56 °C overnight, then frozen at −80 °C for twelve hours followed by an incubation at 56 °C for another twelve hours. The primer pair LCO1490/HCO2198 proposed by Folmer et al. [12] was used to amplify the mitochondrial gene COI, and the PCR was run with the following setup: initial denaturation at 98 °C for 5 min and a five-cycle preamplification (30s at 98 °C, 40s at 45 °C, and 1 min at 72 °C), followed by 35 cycles of 30s at 98 °C, 40s at 55 °C, and 1 min at 72 °C, and a final extension of 5 min at 72 °C. Sequencing of the final product was performed in both forward and reverse directions and edited using Geneious Prime 2024.0.5.

All the sequences were translated into amino acids in Geneious Prime 2024.0.5 to identify any stop codons and then aligned using the MAFFT plugin within Geneious Prime 2024.0.5. The final alignment had a length of 708 bp including undefined nucleotides (N) for some sequences (Dataset S3).

Two datasets were compiled for the analysis: dataset A (96 sequences of *Laelius*) used for species delimitation (Dataset S3); dataset B (97 sequences) used for phylogenetic analysis with one more sequence of species *Gracilepyris rufipes*, which was published by Colombo et al. [1], as an outgroup (Dataset S4). Two different methods were used for species delimitation: the distance-based method Automatic Barcode Gap Discovery (ABGD) and the tree-based method Poisson Tree Process (PTP) [13]. The ABGD method was performed online at https://bioinfo.mnhn.fr/abi/public/abgd/abgdweb.html, accessed on 27 July 2024, applying the K80 model, 30 for Nb bins, and default parameters for the rest. The PTP method was performed online at https://species.h-its.org/ptp/, accessed on 18 July 2024, applying “unrooted” for tree type, 500,000 for “NO. MCMC generations”, and default parameters for the rest. The ML tree for PTP analysis was generated through IQ-TREE-2.3.5-Windows through default parameters [14]. For the phylogenetic analysis, the ML tree was constructed in IQ-TREE version 2.3.5 performed with 1000 ultrafast bootstrap replicates [15] and GTR+G+I, which was chosen as the best-fit substitution model by BIC and AICs calculated in MEGA 11 [16]. The final ML tree was visualized and edited with iTOL [17].

## 3. Results

### 3.1. Species Delimitation

**Figure 1:**
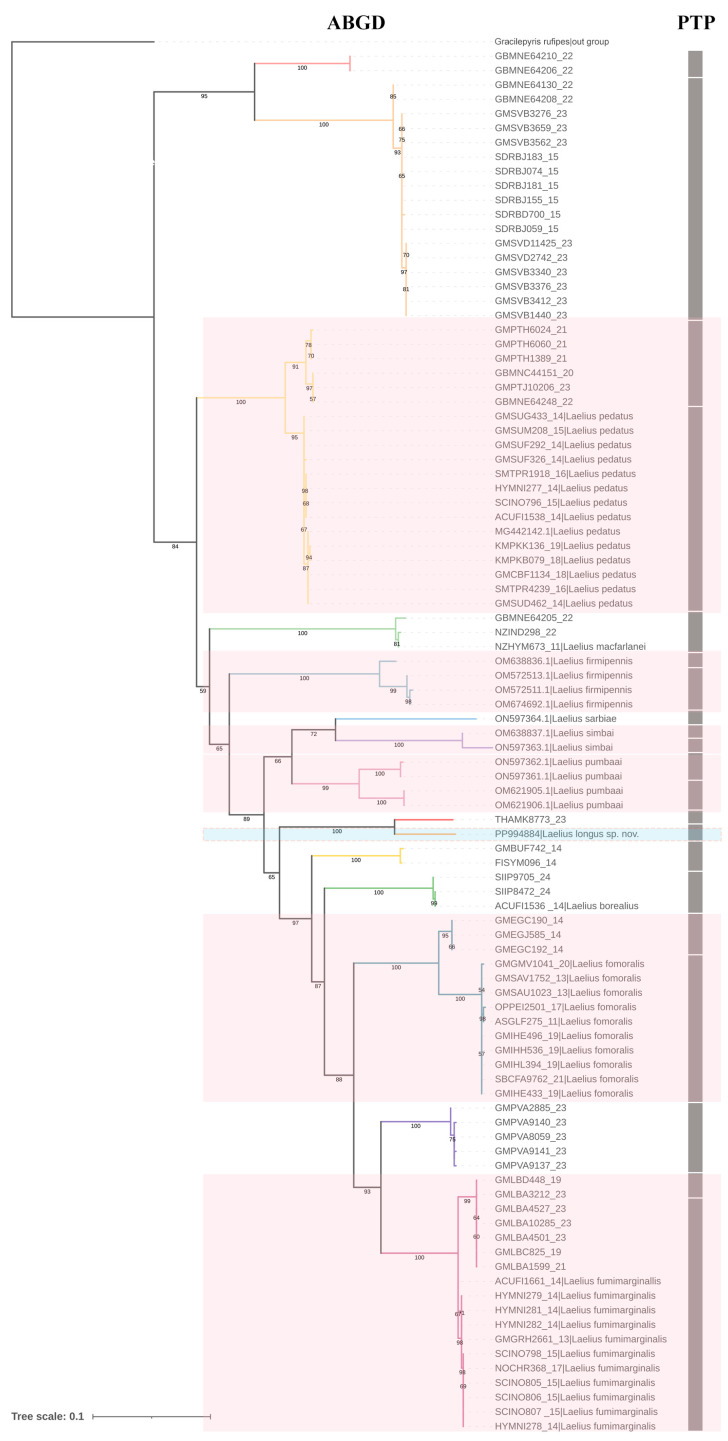
ML phylogenetic tree based on 97 COI sequences highlighting the results of two delimitation analyses in *Laelius*. Bootstrap values are shown below the branches with values greater than 0.50. The delimitation results of ABGD analysis are displayed with branches in different colors, and the results of PTP analysis are displayed with the grey vertical bar on the right. The new species is marked in light blue. The different delimitation results between ABGD and PTP analyses are marked in light pink.

The two delimitation analyses resulted in the pattern shown in Figure 1. The ABGD method matched better with morphological identification and phylogeny analysis than PTP in our study. The 96 COI sequences of *Laelius* were delimited into 15 MOUTs with a barcoding gap of 0.061 (6.1%) by ABGD analysis. The PTP analysis split the morphological species *Laelius pumbaai*, *L. simbai* and *L. firmipennis* into two MOUTs each, resulting in a total of 21 MOUTs. The intraspecific genetic divergence of PTP’s MOUTs ranged from 0 to 1.16418464% (Table S5), which is much lower than that in Marques et al.’s study (0–7%) [5]. Therefore, the PTP analysis in our study may tend to oversplit species. Both the intra- and interspecific genetic divergences among PTP’s delimitation are much lower than previous studies. Despite all of this, the new species *Laelius longus* sp. nov. is supported as a separated species in both two analyses, and the results for species delimitation were consolidated in the ML tree (Figure 1).

### 3.2. Taxonomy

Genus *Laelius* Ashmead, 1893

*Laelius* Ashmead, 1893: 50 [18]. Type species: *Laelius trogogermatis* Ashmead, 1893, by original designation, now regarded as a synonym of *Laelius centratus* (Say, 1836).

*Paralaelius* Kieffer, 1905: 129 [19]. Type species: *Bethylus pedatus* Say, 1836, by subsequent designation by Kieffer, 1914. Synonymized by Muesebeck and Walkley, 1951: 728 [20].

*Allepyris* Kieffer, 1905: 106 [19]. Type species: *Allepyris microneurus* Kieffer, 1905, by original designation. Synonymized by Terayama, 2006: 136 [21].

*Prolaelius* Kieffer, 1905: 251 [22]. Type species: *Paralaelius firmipennis* Cameron, 1905, by original designation. Synonymized by Barbosa and Azevedo, 2011: 254 [23].

Diagnosis. Body and wings with black thick setae; median clypeal lobe well projected; lateral clypeal lobe usually inconspicuous; occipital carina complete; mesoscuto-scutellar suture with foveae connected by sulcus usually smooth; posterior margin incurved.

Host. Mostly larvae of Coleoptera (Dermestidae, Chrysomelidae, Curculionidae) with exception of species *Laelius glossinae*, which was recorded being bred from pupa of *Glossina morsitans* (Diptera: Glossinidae) [3].

Distribution. Cosmopolitan.

#### 3.2.1. Description of New Taxa

**Figure 2:**
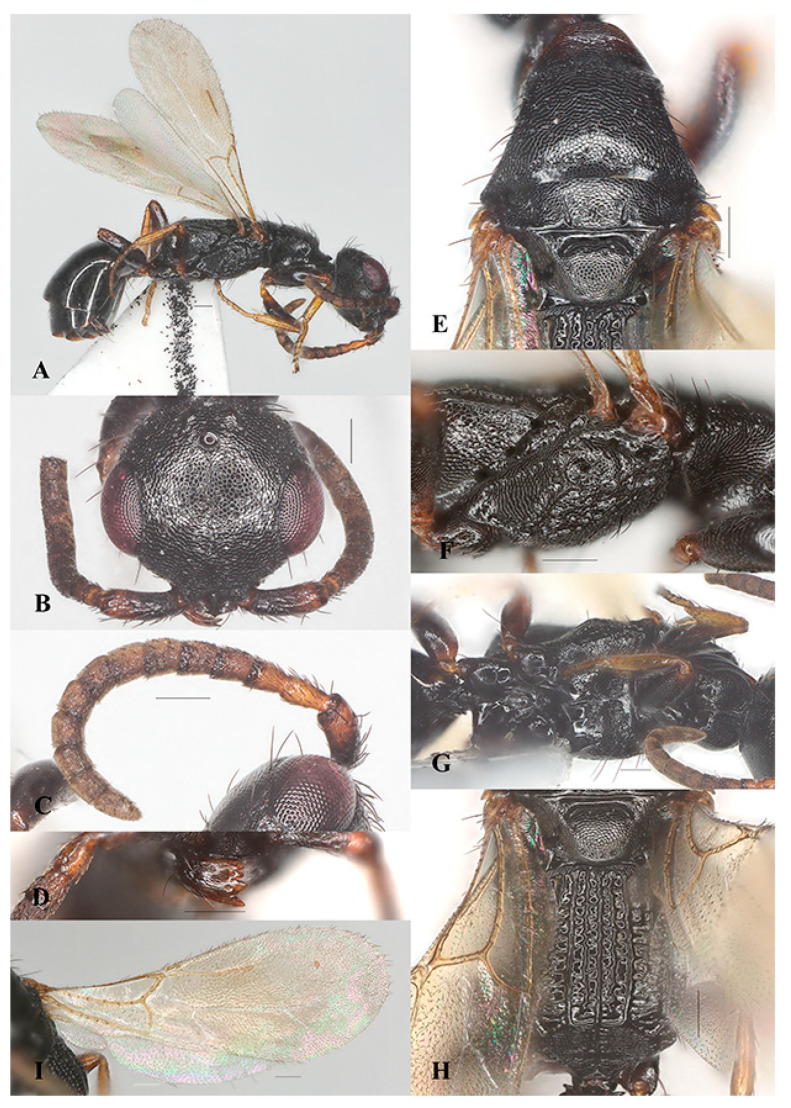
*Laelius longus* Wang, He and Chen sp. nov., holotype, female. (**A**) habitus, lateral view; (**B**) head, antero-dorsal view; (**C**) antenna; (**D**) mandible; (**E**) pronotum and mosonotum, dorsal view; (**F**) mesosoma, lateral view; (**G**) mesosoma, ventral view; (**H**) metapectal-propodeal complex, dorsal view; (**I**) fore wing. Scale bars: 0.15 mm.

*Laelius longus* Wang, He and Chen sp. nov. (Figure 2A–I)

urn:lsid:zoobank.org:act:BE0B2A48-F6EB-48EA-81AE-4C8ACFC620D9

Diagnosis. The new species *Laelius longus* sp. nov. can be recognized by the presence of frontal sulcus (Figure 2B), mesoscutum with notaulus and parapsidal signum (Figure 2E), metapostnotum with five longitudinal carinae extending to transverse posterior carina of metapectal-propodeal complex or nearly so (Figure 2H), fore wing with 2r-rs&Rs vein longer than Rs&M vein, and legs with femora darker than the tibia and tarsi.

This new species is similar to *L. ogmos* Barbosa and Azevedo, 2011, for having frontal sulcus present, fore wing with 2r-rs&Rs vein longer than Rs&M vein, and legs with femora much darker than tibia and tarsi. However, the new species can be distinguished from the latter by having head 0.95 × its width (head as long as wide in *L. ogmos*), frontal sulcus inconspicuous and discrete (conspicuous and continuous in *L. ogmos*), and fore wing with 2r-rs&Rs vein 2.62 × Rs&M vein (2r-rs&Rs vein 2.0 × Rs&M vein in *L. ogmos*).

Description. Holotype. Female. Body length 3.14 mm. Fore wing length 2.08 mm.

Color. Black. Mandible yellowish brown, black basally. Antenna yellowish brown, scape blackish brown except apically. Legs yellow to yellowish brown; coxae and femora (except basally and apically) dark brown. Fore wing hyaline, tinged with light yellow; veins and pterostigma yellow.

Head (Figure 2B–D). Head wider than long, LH 0.95 × WH. Mandible with five teeth, ventralmost two much larger (Figure 2D). Median clypeal lobe subtrapezoidal, anterior margin with small median tooth; medial clypeal carina present, not exceeding antennal foramen. Antennal flagellomeres with short appressed setae; scape, pedicel and flagellomere I with black thick setae; pedicel longer than flagellomere I, length of pedicel 1.3 × length of flagellomere I (Figure 2C). Frons distinctly coriaceous with shallow punctures; frontal sulcus weak and discontinuous; WF 0.6 × WH. Eye glabrous, WF 1.38 × LE. Vertex distinctly coriaceous with shallow punctures, sides of head posterior to eyes converging posterad; anterior ocellus posterior to supra-ocular line; POL 2.12 × AOL, OOL 1.27 × WOT; vertex crest slightly outcurved; occipital carina complete. Gena coriaceous, medioccipito-genal line carinate.

Mesosoma (Figure 2E–G). Dorsal pronotal area distinctly coriaceous with shallow punctures, median length 0.5 × width along posterior pronotal margin; posterior pronotal sulcus present, discontinuous; pronotal flange and lateral pronotal area coriaceous. Mesoscutum flat and coriaceous, apical half with punctures; width of mesoscutum between tergulae 2.82 × its median length; median mesonotal sulcus absent; notaulus incomplete, converging posterad; parapsidal signum incomplete. Mesoscutellum coriaceous with sparse punctures. Metapostnotum with median metapostnotal carina, first metapostnotal lateral carinae nearly complete, second metapostnotal lateral carinae complete; third metapostnotal lateral carina incomplete, present on basal one-third; metapostnotal–propodeal carina incomplete, present on basal one-third; paraspiracular carina and lateral marginal carina of metapectal-propodeal complex complete; area between longitudinal carinae with short transverse carinae; transverse posterior carina of metapectal-propodeal complex complete and incurved; propodeal declivity coriaceous, median carina complete, lateral carina weak; lateral surface of propodeum coriaceous, metapleural line with three foveae. Mesopectus strongly coriaceous; lateral surface of mesopectus with subalar impression, anterior subalar and posterior subalar pits present, upper mesopleural fovea closed, two closed lower mesepimeral fovea present; ventral surface of mesopectus coriaceous.

Wings. Macropterous, fore wing with 2r-rs&Rs vein distinctly longer than Rs&M vein, length of 2r-rs&Rs vein 2.62 × length of Rs&M vein. Hind wing with three distal hamuli (Figure 2I).

Metasoma. Metasomal terga I–II polished, other terga weakly coriaceous basally; metasomal sternum I coriaceous with ‘Y’-shaped carina, metasomal sternum II polished with sparse punctures.

Male. Unknown.

Material examined. Holotype, ♀, China: Yunnan, Xishuangbanna, Menghai County, Bulangshan Country, Banzhang Village, 21.751° N, 100.361° E, elev. 1706 m, Malaise trap, 9.VII–13.XI.2021 (No. 202400001).

Distribution. China (Yunnan).

Etymology. The specific epithet *longus*, which means “long”, is the Latin word referring to the new species with the 2r-rs&Rs vein of the fore wing distinctly longer than the Rs&M vein.

Remarks. Among the six *Laelius* species recorded in China, *Laelius longus* sp. nov. is the first and the only one species with the 2r-rs&Rs vein distinctly longer than the Rs&M vein.

#### 3.2.2. Newly Recorded Species from China

##### *Laelius naniwaensis* Terayama, 2006 (Figure 3A–G and Figure 4A–I)

*Laelius naniwaensis* Terayama, 2006: 137 [21].

Material examined. China: 36♀, Hunan Pro., reared from larvae of *Thaumaglossa* sp., 19.VI.1983, Xinwang Tong leg. (No. 200610417, 200610419–200610431, 200610434, 200610436–442, 200610444–458); 3♂, same information as ♀, No. 200610416, 200610429, 200610443; 1 ♂, Jiangsu Prov., Nanjing, 5.XI.1989, Yunzhen Sun leg. (No. 20004708).

**Figure 3:**
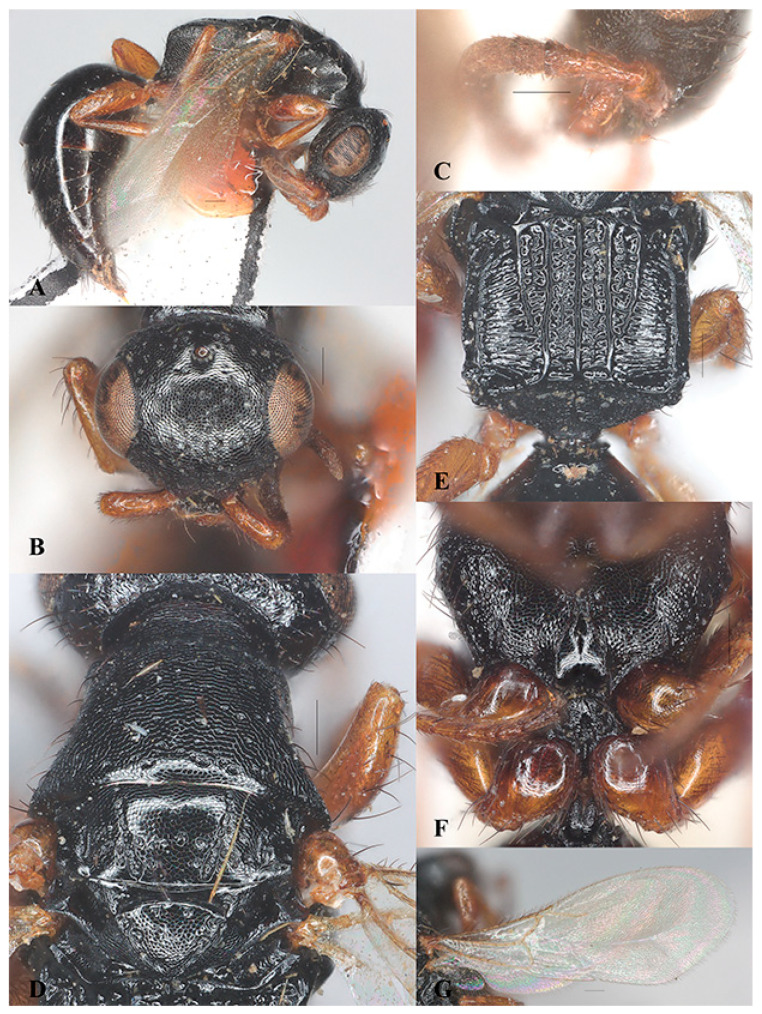
*Laelius naniwaensis* Terayama, 2006, female. (**A**) Habitus, lateral view; (**B**) head, antero-dorsal view; (**C**) antenna; (**D**) pronotum and mosonotum, dorsal view; (**E**) metapectal-propodeal complex, dorsal view; (**F**) mesosoma, ventral view; (**G**) fore wing. Scale bars: 0.15 mm.

**Figure 4:**
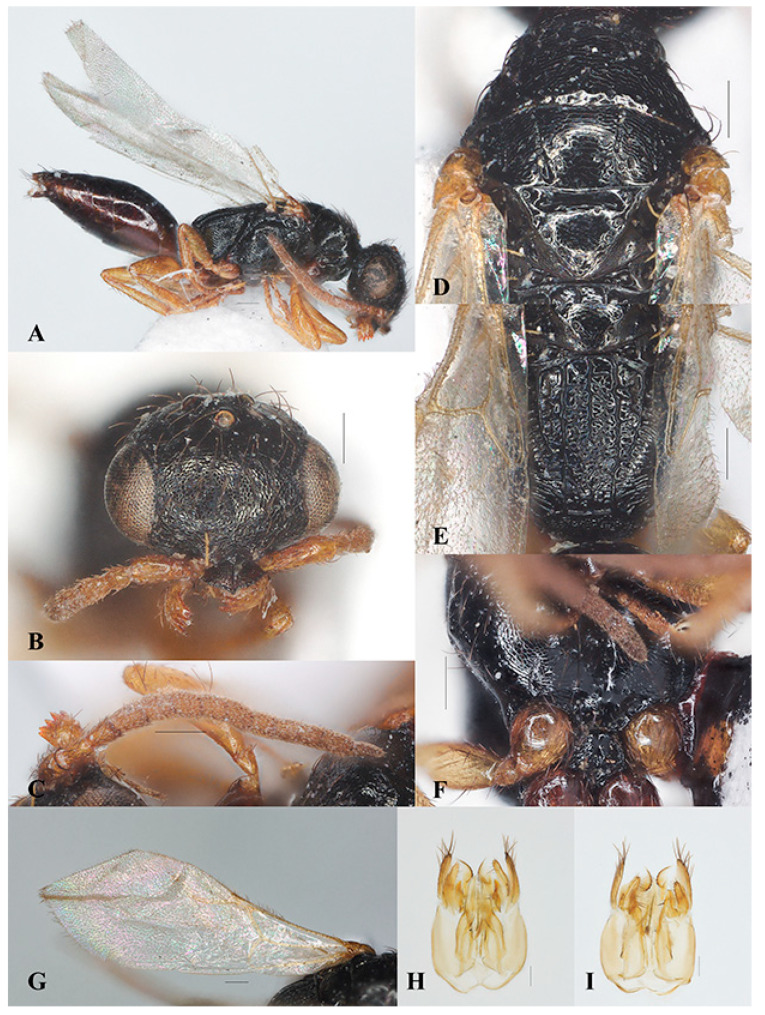
*Laelius naniwaensis* Terayama, 2006, male. (**A**) habitus, lateral view; (**B**) head, antero-dorsal view; (**C**) antenna; (**D**) pronotum and mosonotum, dorsal view; (**E**) metapectal-propodeal complex, dorsal view; (**F**) mesosoma, ventral view; (**G**) fore wing; (**H**) genitalia, dorsal view; (**I**) genitalia, ventral view. Scale bars: 0.15 mm.

Diagnosis. Legs are yellowish brown (Figure 3A and Figure 4A). Head is distinctly wider than long with vertex crest distinctly outcurved (Figure 3B and Figure 4B). Mesoscutum with notaulus complete but discontinuous. Metapostnotum with three distinct carinae extending to transverse posterior carina of metapectal-propodeal complex in females (Figure 3E); the carinae are weaker in males (Figure 4E). Ventral surface of mesopectus with pair of distinct elevations near mesocoxa in females (Figure 3F), weaker in males (Figure 4F). Fore wing with the 2r-rs&Rs vein slightly longer than the Rs&M vein (Figure 3G and Figure 4G). Male genitalia are shown in Figure 4H,I.

Distribution. China (Jiangsu, Hunan), Japan.

**Figure 5:**
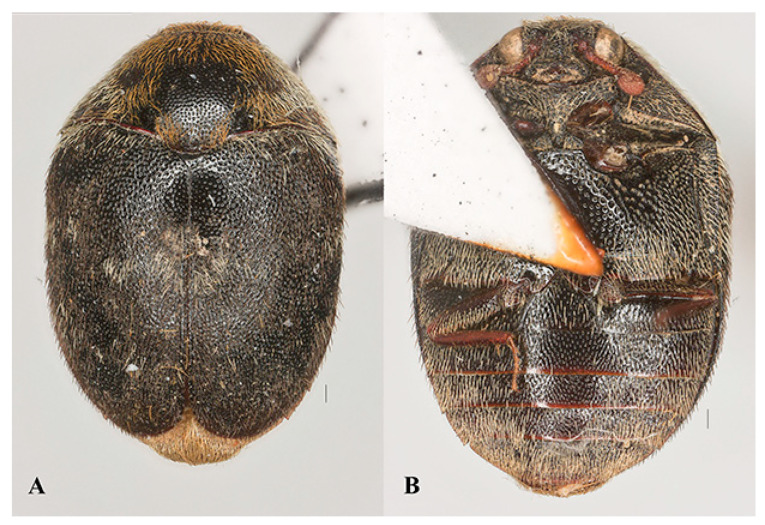
*Thaumaglossa* sp. (**A**) habitus, dorsal view; (**B**) habitus, ventral view. Scale bars: 0.15 mm.

Host. *Thaumaglossa ruficapillata* (Coleoptera, Dermestidae) [21], *Thaumaglossa* sp. (Coleoptera, Dermestidae) (Figure 5A,B).

Remarks. The species *Laelius naniwaensis* is the first record of *Laelius* in China associated with dermestid hosts. Some species of the dermestid genus *Thaumaglossa* have been recorded as inquiline and predators of the mantis oothecae [24,25]. The mantis ootheca, which is a sclerotized protein-based layer produced by the female mantis, is not only an important structure for mantid breeding but is also sometimes an integral ingredient of traditional Chinese herbal medicine. Therefore, *L. naniwaensis* could be a potential biocontrol agent for agroforestry and store product pests.

##### *Laelius nigrofemoratus* Terayama, 2006 (Figure 6A–F)

*Laelius nigrofemoratus* Terayama, 2006: 140 [21].

Type material examined. Paratype: Japan: 1♀, Ishikawa Prefecture, Ohsugidani, Shiramine-mura, 30.VII.1991, I. Togashi leg.

Other material examined. China: 1♀, Hunan, Huaihua, Xupu County, 31.VII.1978, Lexiang Ni leg. (No. 20044994).

**Figure 6:**
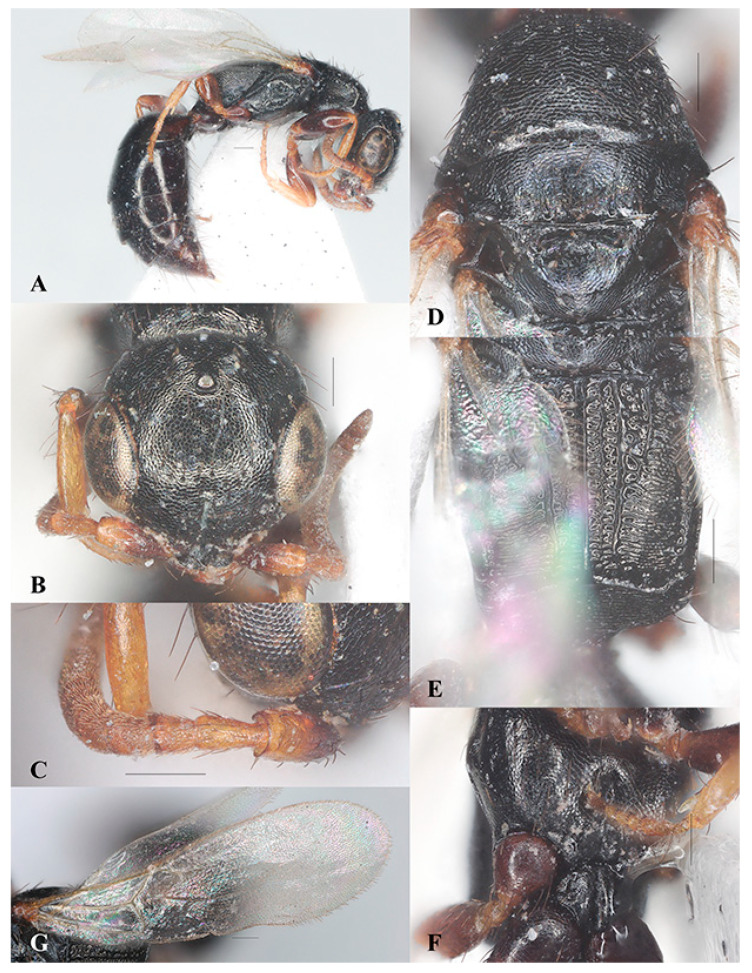
*Laelius nigrofemoratus* Terayama, 2006, female. (**A**) Habitus, lateral view; (**B**) head, antero-dorsal view; (**C**) antenna; (**D**) pronotum and mosonotum, dorsal view; (**E**) metapectal-propodeal complex, dorsal view; (**F**) mesosoma, ventral view; (**G**) fore wing. Scale bars: 0.15 mm.

Diagnosis. Legs with coxae and femora darker than tibiae and tarsi (Figure 6A). Head as long as wide (Figure 6B). Mesoscutum with notaulus complete (Figure 6D). Metapostnotum with five longitudinal carinae nearly extending to transverse posterior carina of metapectal-propodeal complex, of which the first metapostnotal lateral carina is much weaker (Figure 6E). Ventral surface of mesopectus with pair of elevations near mesocoxa (Figure 6F) but much weaker than in *L. naniwaensis* (Figure 3F). Fore wing with 2r-rs&Rs vein shorter than Rs&M vein (Figure 6G).

Distribution. China (Hunan), Japan.

Host. *Anthrenus* sp. (Coleoptera, Dermestidae) [21].

##### *Laelius yamatonis* Terayama, 2006 (Figure 7A–F)

*Laelius yamatonis* Terayama, 2006: 141 [21].

Type material examined. Paratype: Japan: 1♀, Tokyo, Sakuragaoka, Setagaya-ku, 22.XI.1972, Y. Yoshikawa leg.

Other material examined. China: 1♀, Zhejiang, Hangzhou, 21.VII.1957, Junhua He leg. (No. 5735.2); 1♀, same locality, 5.XI.1983, Junhua He leg. (No. 835237); 1♀, same locality, VIII.1988, Junhua He leg. (No. 893613); 1♀, Zhejiang, Hangzhou, Tianmu Mountain, 16.X.1982, Junhua He leg. (No. 826346); 4♀, Zhejiang, Lishui, Songyang County, 24.8° N, 119.4° E, 9.VII–13.XI.1980, Hanlin Chen leg. (No. 897046, 897057, 897063, 907839); 1♀, Hainan, Jianfeng Ling, Sky Pond, 9.V.2008, Jingxian Liu leg. (No. 200800148); 1♀, Guizhou, Guiyang, VII.1982, Xuepei Song leg. (No. 835044).

**Figure 7:**
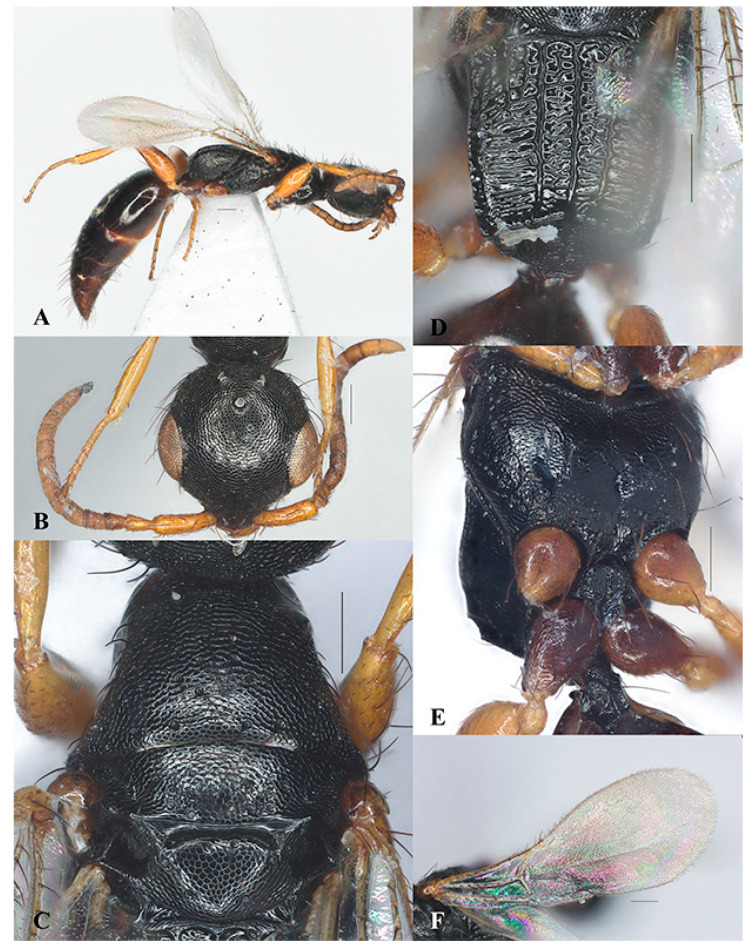
*Laelius yamatonis* Terayama, 2006, female. (**A**) Habitus, lateral view; (**B**) head (antero-dorsal view) and antenna; (**C**) pronotum and mosonotum, dorsal view; (**D**) metapectal-propodeal complex, dorsal view; (**E**) mesosoma, ventral view; (**F**) fore wing. Scale bars: 0.15 mm.

Diagnosis. Legs yellowish brown (Figure 7A). Head slightly longer than wide (Figure 7B). Mesoscutum with notaulus incomplete and inconspicuous (Figure 7C). Metapostnotum with three longitudinal carinae that extend to transverse posterior carina of metapectal-propodeal complex (Figure 7D). Ventral surface of mesopectus without pair of elevations near mesocoxa (Figure 7E). Fore wing with the 2r-rs&Rs vein shorter than Rs&M vein (Figure 7F).

Distribution. China (Zhejiang, Hainan, Guizhou), Japan.

Host. *Anthrenus verbasci* (Coleoptera, Dermestidae) [21].

Key to the Chinese species of *Laelius* Ashmead, 1893

1. Female·······························································································2
  Male··································································································6
2. Frons with frontal sulcus present; fore wing with 2r-rs&Rs vein 2.62 × Rs&M vein································································*L. longus* sp. nov.
  Frons without frontal sulcus; fore wing with 2r-rs&Rs vein shorter or slightly longer than Rs&M vein···························································3
3. Mandible with four teeth; second metapostnotal lateral carina weak and not extending to transverse posterior carina of metapectal-propodeal complex······················································································································*L. jilinensis*
  Mandible with five teeth; second metapostnotal lateral carina developed and extending to transverse posterior carina of metapectal-propodeal complex···4
4. Head distinctly wider than long; fore wing with 2r-rs&Rs vein slightly longer than Rs&M vein·······································································*L. naniwaensis*
  Head as long as or longer than wide; fore wing with 2r-rs&Rs vein shorter than Rs&M vein·································································································5
5. Head as long as wide; notaulus complete; first metapostnotal lateral carina nearly extending to transverse posterior carina of metapectal-propodeal complex; legs with color of femora much darker than tibiae and tarsi·······················································································································*L. nigrofemoratus*
  Head longer than wide; notaulus incomplete, present as fovea near posterior margin of mesoscutum; first metapostnotal lateral carina weak, extending to at most two-thirds of metapostnotum; legs with femora, tibiae and tarsi in same color·······························································································*L. yamatonis*
6. Notaulus incomplete; fore wing with 2r-rs&Rs vein shorter than Rs&M vein; posterior margin of hypopygium nearly straight····························*L. sinicus*
  Notaulus complete; fore wing with 2r-rs&Rs vein longer than Rs&M vein; posterior margin of hypopygium distinctly incurved·············*L. naniwaensis*

## 4. Discussion

The variation of the main morphological characters among *Laelius* has been systematically discussed by Marques et al. [5] and there are four patterns in the length of the 2r-rs&Rs vein of the fore wing in *Laelius*: absent, stub, short, and long. The pattern of the 2r-rs&Rs vein of fore wing is usually stable between conspecific males and females, e.g., *Laelius centratus* (Say, 1836), *L. naniwaensis* Terayama, 2006, *L. pedatus* (Say, 1836), *L. simplex* Evans, 1978, *L. voracis* Muesebeck, 1939, and *L. yamatonis* Terayama, 2006. However, the conspecific male–female association remains a challenge because of the presence of sexual dimorphism among *Laelius*, e.g., the carinae on the dorsal surface of metapectal-propodeal complex are usually less developed in males than in females (Figure 3E and Figure 4E), or the females are micropterous while the males are macropterous, e.g., *Laelius pumbaai*. Our phylogenetic analysis concerning *Laelius* underlined that COI sequences could be a reliable delimitation marker for this genus since monophyletic status at the species level was confirmed in all taxa (Figure 1). Nonetheless, the application of different delimitation approaches may result in different numbers of putative species or MOUTs [26]. In the present study, the 96 COI sequences dataset of *Laelius* was split into 15 MOUTs by ABGD but into 21 MOUTs by PTP. In our case, the distance-based method ABGD performed better than the tree-based method PTP in terms of congruence between reconstructed MOTUs and morphological taxonomy. For instance, the sequences labeled *Laelius firmipennis*, *L. simbai*, and *L. pumbaai* were supported as one hypothesized species, while all of them were split into two MOUTs in PTP analysis. However, we also noticed the unusual genetic divergence within *Laelius pumbaai*. This species was split into two clades, each of them with a genetic distance less than 0.0018050542, while the genetic distance between the two clades was 0.054438537 (Table S5). It is true that the efficiency of molecular species delimitation could be affected by various factors, e.g., the number of haplotypes per species, the geographic distance among conspecific collection localities, and the taxonomic rank [27]. Therefore, further study with a larger number of specimens and the use of multiple genes through various delimitation methods is necessary to establish a practical DNA barcode library of *Laelius*.

## Data Availability

DNA data generated in this study are available in Genbank under accession numbers (Table S1). Other public DNA data downloaded from BOLD and Genbank are available under corresponding accession numbers (Table S2).

## Supplementary Materials

The following supporting information can be downloaded at https://www.mdpi.com/article/10.3390/insects15080627/s1. Table S1: List of specimens used for DNA extraction; Table S2: List of terminal taxa information used for molecular analysis, with BOLD and Genbank accession numbers; Dataset S3: COI alignment (708 bp) of 96 *Laelius* sequences; Dataset S4: COI alignment (708) of 97 sequences for phylogenetic analysis; Table S5: Pairwise genetic distance of *Laelius* based on 96 COI sequences under K2P model; Delimitation Result 1 S6: ABGD results based on Dataset S3; Delimitation Result 2 S7: PTP results based on Dataset S3; Treefile S8: Maximum likelihood tree of *Laelius* (Dataset S4) based on GTR+G+I model with *Gracilepyris rufipes* as outgroup.
