# Supplementary figures and images for: Revision of the Genus Laelius (Hymenoptera, Chrysidoidea, Bethylidae) from China
Source: Insects. 2024 Aug 20;15(8):627. doi: 10.3390/insects15080627 (PMC11355002; doi:10.3390/insects15080627)

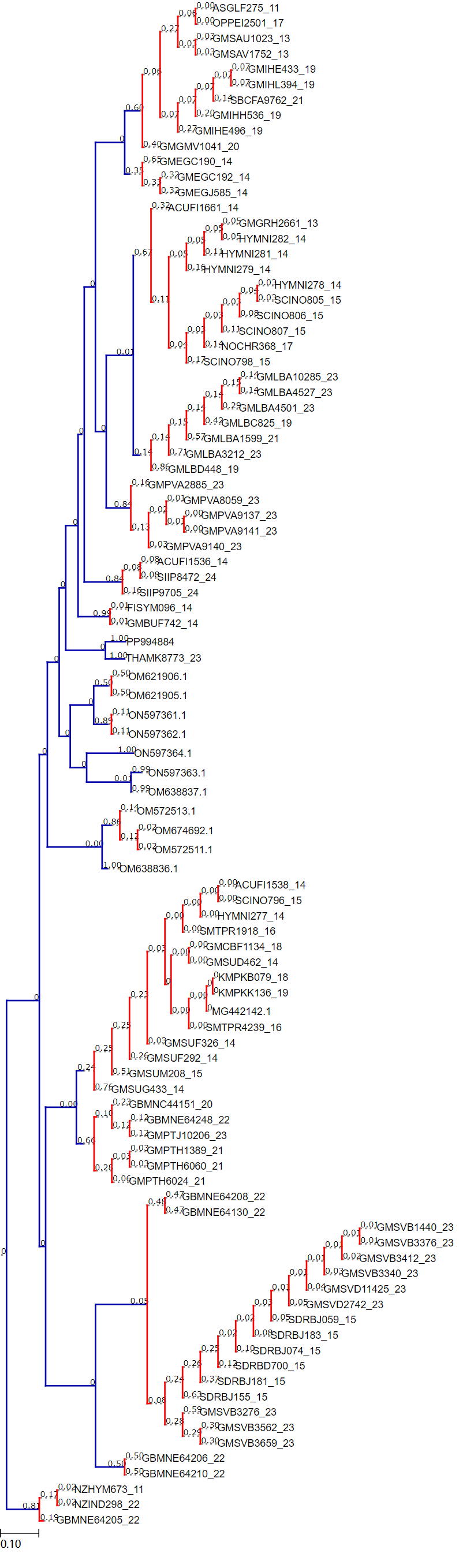

Supplement: Supplementary file 1 [file insects-15-00627-s001.zip › supplementary materials/S7_Delimitation Result2.png]
